# Increasing the efficiency of CRISPR‐Cas9‐VQR precise genome editing in rice

**DOI:** 10.1111/pbi.12771

**Published:** 2017-08-05

**Authors:** Xixun Hu, Xiangbing Meng, Qing Liu, Jiayang Li, Kejian Wang

**Affiliations:** ^1^ State Key Laboratory of Rice Biology China National Rice Research Institute Chinese Academy of Agricultural Sciences Hangzhou China; ^2^ State Key Laboratory of Plant Genomics and National Center for Plant Gene Research (Beijing) Institute of Genetics and Developmental Biology Chinese Academy of Sciences Beijing China; ^3^ University of Chinese Academy of Sciences Beijing China

**Keywords:** CRISPR‐Cas9, VQR, genome editing, efficiency, rice

## Abstract

Clustered regularly interspaced short palindromic repeats‐associated protein 9 (CRISPR‐Cas9) is a revolutionary technology that enables efficient genomic modification in many organisms. Currently, the wide use of *Streptococcus pyogenes* Cas9 (SpCas9) primarily recognizes sites harbouring a canonical NGG protospacer adjacent motif (PAM). The newly developed VQR (D1135V/R1335Q/T1337R) variant of Cas9 has been shown to cleave sites containing NGA PAM in rice, which greatly expanded the range of genome editing. However, the low editing efficiency of the VQR variant remains, which limits its wide application in genome editing. In this study, by modifying the single guide RNA (sgRNA) structure and strong endogenous promoters, we significantly increased the editing efficiency of the VQR variant. The modified CRISPR‐Cas9‐VQR system provides a robust toolbox for multiplex genome editing at sites containing noncanonical NGA PAM.

## Introduction

The clustered regularly interspaced short palindromic repeats‐associated protein 9 (CRISPR‐Cas9) system with a single guide RNA (sgRNA) has exhibited powerful capabilities for genome editing in eukaryotes and is widely used for investigating the function of genes or rapidly obtaining new alleles (Cong *et al*., 2013; Hsu *et al*., 2014; Mali *et al*., 2013; Shan *et al*., 2013). Editing efficiency is affected by many factors, including the low GC contents of the guide sequence, secondary structures of target sequences (Ma *et al*., 2015b; Wang *et al*., 2014; Zhang *et al*., 2014) and continuous A or T on the guide sequence (Dang *et al*., 2015). Thus, although the CRISPR‐Cas9 provides a robust system for genome editing, it remains inefficient or hardly applicable in many genomic sites. To increase the editing efficiency in animal cells or embryos, pre‐assembled Cas9 protein–gRNA ribonucleoproteins (RNPs) are frequently used. Recently, it was found that nonhomologous single‐stranded DNA could stimulate a disruption frequency, and that the sgRNA structure could be optimized to increase the efficiency of the CRISPR‐Cas9 system in mammalian cells (Dang *et al*., 2015; Kim *et al*., 2014; Lin *et al*., 2014; Richardson *et al*., 2016a,b). In addition, RNA‐guided TevCas9 dual nuclease was developed, which could increase the efficiency of the CRISPR‐Cas9 system by creating a deletion of small fragments (Wolfs *et al*., 2016). In regard to plants, the main strategy employed to increase the editing efficiency of the CRISPR‐Cas9 system is to use strong promoters to drive high expression of Cas9 and to avoid usage of a low‐scored guide sequence (Ma *et al*., 2015b; Shan *et al*., 2013; Zhang *et al*., 2014).

Currently, the robust and widely used *Streptococcus pyogenes* Cas9 (SpCas9) requires the sites containing NGG protospacer adjacent motifs (PAMs) (Hsu *et al*., 2014). To expand the range of CRISPR‐Cas9 genome editing, many orthologs or variants of Cas9 proteins were screened out, such as *S. thermophilus* CRISPR3 Cas9 for NGGNG PAMs (Horvath *et al*., 2008), *S. thermophilus* CRISPR1 Cas9 for NNAGAAW PAMs (Deveau *et al*., 2008), *Neisseria meningitidis* Cas9 for NNNNGATT PAMs (Zhang *et al*., 2013), and VQR variants of SpCas9 for NGA PAMs and VRER variants for NGCG PAMs (Kleinstiver *et al*., 2015). In plants, the VQR and VRER variants of Cas9 for genome editing have been developed, which greatly extended the range of genome editing (Hu *et al*., 2016). However, the efficiency of these variants remains low compared with that of wild‐type (WT) Cas9, which limits its wide application for genome editing.

In this study, we aimed to increase the editing efficiency of the CRISPR‐Cas9‐VQR system. By modifying the sgRNA structure and expressing VQR variants with strong endogenous promoters, we dramatically increased the editing efficiency of VQR variants in rice. Thus, the newly modified CRISPR‐Cas9‐VQR system is particularly suitable for efficient genome editing in NGA PAMs.

## Results

**Figure 1:**
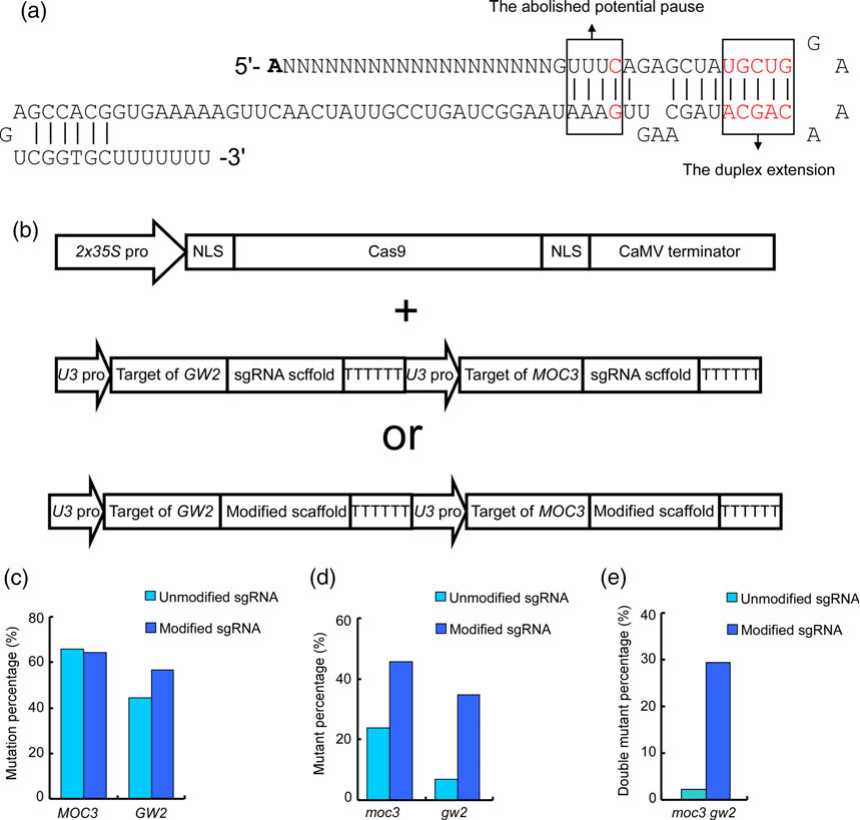
Modified single guide RNAs (sgRNAs) increased the efficiency of the clustered regularly interspaced short palindromic repeats‐associated protein 9 (CRISPR‐Cas9) system. (a) Schematic representation of modified sgRNAs. The replaced or introduced nucleotides are highlighted in red. These mutations abolish a potential transcription pause site and add a duplex extension for sgRNA stability. (b) The architecture of vectors in the CRISPR‐Cas9 system. The Cas9 protein attached to the nuclear localization signal (NLS) is driven by the *2x35S* promoter. Both unmodified and modified sgRNAs are expressed by the *U3* promoter. (c) The mutation rates at *MOC3* and *GW2*. The modified sgRNA and unmodified sgRNA are represented in dark blue and light blue, respectively. (d) The proportion of mutants (biallelic or chimeric mutants) of *moc3* and *gw2*. By modifying sgRNAs, the proportion of mutants increased approximately twofold or fivefold at *MOC3* and *GW2*, respectively. (e) The proportion of *moc3 gw2* double mutants. Modified sgRNAs dramatically increased (approximately 13‐fold) the proportion of double mutants.

We first modified the sgRNA structure to that in mammalian cells (Dang *et al*., 2015) and tested whether the modification is able to increase the editing efficiency of CRISPR/Cas9 in plants. The previously widely used gRNAs contain continuous sequences of Ts, which are the pausing signals for RNA polymerase III and efficiently reduce transcription. We replaced the fourth T in the sequence of Ts with C to eliminate the pausing signal. In addition, five pairs of bases were added into the distal duplex of the sgRNA to enhance its stability or binding to the Cas9 protein (Dang *et al*., 2015) (Figure 1a). We screened the rice genome and selected two sites containing NGG PAMs in the *MONOCULM 3* (*MOC3*) and *GRAIN WIDTH ON CHROMOSOME 2* (*GW2*) genes for genome editing (Lu *et al*., 2015; Song *et al*., 2007). Both guide sequences were calculated to be low‐scored sites (Ma *et al*., 2016; Xu *et al*., 2015) (Table S1). Furthermore, the guide sequence of *GW2* also contains low GC (31.58%) and a continuous sequence close to the PAM, both of which would affect the editing efficiency. The guide sequences were cloned into a modified and unmodified sgRNA, respectively. The two guide sequences using the same sgRNAs were then assembled into one vector (Figure 1b). The constructed vectors were then transferred into rice by an *Agrobacterium*‐mediated method (Hiei *et al*., 1994). A total of 88 and 92 independent transgenic lines using unmodified and modified sgRNAs were obtained, respectively. By DNA sequencing, we found that all target sites harboured mutations with different mutation rate (Tables S2 and S3). The mutation rate at *MOC3* (from 65.91% to 64.13%) remained stable, whereas that at *GW2* increased from 44.32% to 56.52%. As for the frequency of mutants (biallelic or chimeric mutants), the mutant proportion at the *MOC3* site almost doubled, increasing from 23.86% to 45.65%. However, the mutant proportion at the *GW2* site increased more than fivefold, from 6.82% to 34.78% (Figure 1c, d and Table S3). Furthermore, the proportion of *moc3 gw2* double mutants dramatically increased by approximately 13‐fold (from 2.27% to 29.35%) (Figure 1e and Table S4). These results indicate that the modified sgRNAs are functional in promoting the efficiency of Cas9 at sites containing NGG PAMs in plants.

**Figure 2:**
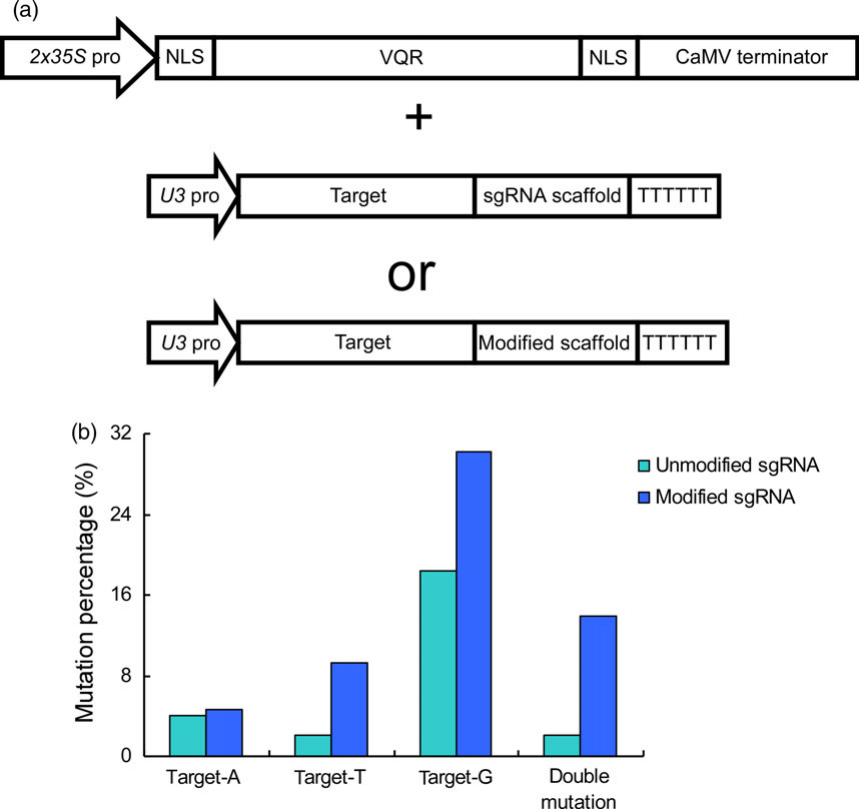
Efficiency of the CRISPR‐Cas9‐VQR system is promoted by modified sgRNAs. (a) The architecture of vectors in the CRISPR‐Cas9‐VQR system. The VQR variants and sgRNAs are expressed by the *2x35S* and *U3* promoters, respectively. The binary vectors are constructed using one of the two sgRNAs. (b) The proportions of single mutations and double mutations. A, T and G, respectively, indicate the base following the NGA PAM. The unmodified sgRNA and modified sgRNA are represented in light blue and dark blue, respectively.

We next investigated whether the modified sgRNAs promote the editing efficiency at NGA PAM sites in the CRISPR‐Cas9‐VQR system. The guide sequence, which has been shown to simultaneously edit three genomic sites harbouring NGA PAMs (Hu *et al*., 2016), was selected. These sites were distinguished by a different base (A, T and G) behind the NGA PAMs. We ligated the guide sequence into the modified sgRNA, which was then assembled into the vector expressing VQR variants (Figure 2a). The resulting vector was subsequently used for genetic transformation, and a total of 43 transgenic lines were obtained. By sequencing all three target sites, we analysed the editing efficiencies and compared the data with those obtained from unmodified sgRNA. By using the modified sgRNAs, the mutation rate at target‐A increased marginally from 4.08% to 4.65%, whereas at target‐T, the rate dramatically quintupled, increasing from 2.04% to 9.30%, and at target‐G, the rate nearly doubled, increasing from 18.37% to 30.23% (Figure 2b, Tables S5 and S6). We then analysed the plants harbouring double mutations and found that the frequency of double mutations increased sharply from 2.04% to 13.95% using the modified sgRNA (Figure 2b and Table S7). These results indicate that the modified sgRNAs are effective in improving the efficiency of the CRISPR‐Cas9‐VQR system.

**Figure 3:**
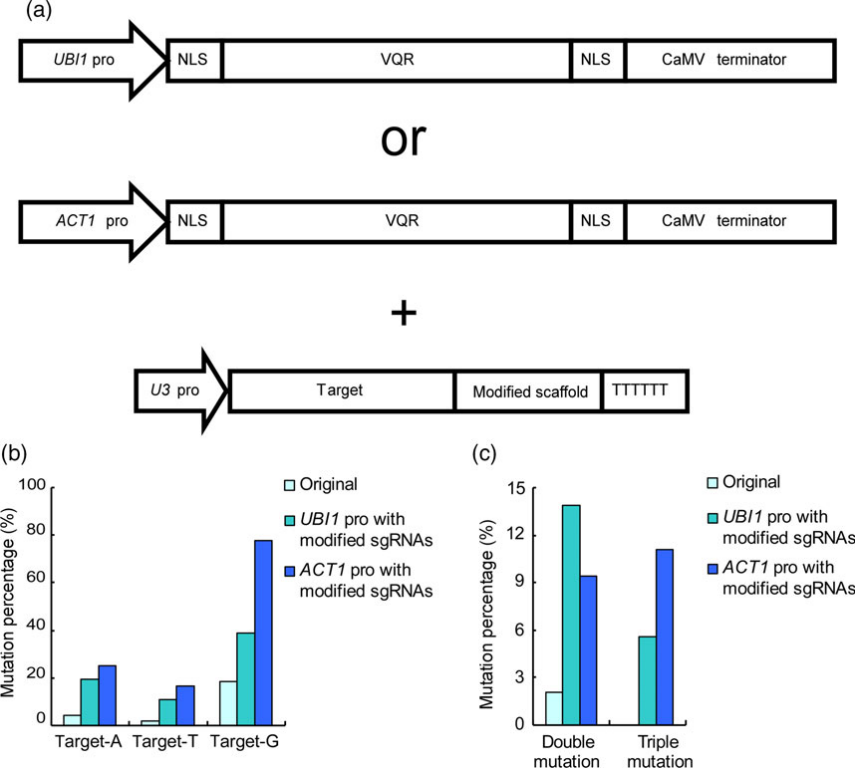
Efficiency of the CRISPR‐Cas9‐VQR system is further increased by the *UBI1* and *ACT1* promoters. (a) The architecture of vectors with different promoters. The promoters of VQR variants are replaced by *UBI1* or *ACT1*. The modified sgRNAs are expressed with *U3* promoters. (b) The mutation rate of the CRISPR‐Cas9‐VQR system using different promoters. The efficiency of the CRISPR‐Cas9‐VQR system is greatly increased by using strong promoters and modified sgRNAs. The system with the *UBI1* promoter increases the mutation rate by an average of approximately fourfold, whereas that with *ACT1* increases the mutation rate by an average of approximately sixfold. (c) The proportion of double and triple mutations using different systems. The proportions of double or triple mutations are dramatically increased. The triple mutations are undetected in the original system.

In our previous studies, we utilized a *2x35S* promoter to drive the expression of VQR protein (Hu *et al*., 2016). To further increase the efficiency of the CRISPR‐Cas9‐VQR system, we replaced the *2x35S* promoter with two endogenous promoters, namely rice *UBIQUITIN1* (*UBI1*) and *ACTIN1* (*ACT1*), respectively (McElroy *et al*., 1990; Wang *et al*., 2000) (Figures S1 and S2). For comparison, the same guide sequence described above was used further for genome editing. After vector construction (Figure 3a) and genetic transformation, 36 transgenic lines using the *UBI1* or *ACT1* promoter were obtained, respectively (Tables S8 and S9). We sequenced the target sites of all transgenic plants and compared the editing efficiency with that obtained by using unmodified sgRNAs and the *2x35S* promoter. When the *UBI1* promoter and the modified sgRNA were co‐applied, the mutation rate at target‐A increased from 4.08% to 19.44%, whereas that at target‐T increased from 2.04% to 11.11% and that at target‐G increased from 18.37% to 38.89% (Figure 3b and Table S10). Furthermore, eight lines were detected to possess biallelic mutations at target‐G sites, which were not found using the original system (Tables S8 and S10). Moreover, the proportion of lines containing double and triple mutations increased from 2.04% to 19.44% (Figure 3c and Table S6).

When the *ACT1* promoter and modified sgRNA were used, the editing efficiency at target‐A increased approximately sixfold from 4.08% to 25.00%, that at target‐T increased approximately eightfold from 2.04% to 16.67%, and that at target‐G increased approximately fourfold from 18.37% to 77.78% (Figure 3b and Table S10). In addition, more than half (52.78%) of transgenic plants were identified as biallelic mutants (Figure 3 and Table S10). Moreover, the proportion of transgenic lines harbouring double and triple mutations increased by approximately 15‐fold from 2.04% to 30.56% (Figure 3c and Table S6). The results imply that both *UBI1* and *ACT1* promoters can significantly promote the editing efficiency of the CRISPR‐Cas9‐VQR system. In particular, the *ACT1* promoter is the most efficient among the three promoters in elevating the editing efficiency of the CRISPR‐Cas9‐VQR system (Figure 3b and Table S10).

A previous study of the CRISPR‐Cas9 system indicated that increased efficiency would raise the risk of off‐targeting effects (Singh *et al*., 2016). To investigate whether similar patterns exist in the CRISPR‐Cas9‐VQR system, we screened the rice genome and selected four sites for analysis. All four sites contained only one mismatch with the target sequence (Table 1). The mismatch was located at the 19‐, 17‐, 15‐ or 6‐base pair (bp) position from NAG PAMs, respectively. By sequencing all these sites in the modified lines, we found that off‐targeting events occurred at VQR‐off‐1, VQR‐off‐2 and VQR‐off‐3 sites, but not at VQR‐off‐4 sites. In addition, the off‐targeting events showed an obvious preference (VQR‐off‐1 > VQR‐off‐2 > VQR‐off‐3) in each line (Table 1, Figure S3 and Table S11). We also compared the off‐targeting events between lines showing different editing efficiency and found that with the increased editing efficiency, the frequency of off‐targeting events increased accordingly (Figure S3).

**Table 1:** The off‐target events in the CRISPR‐Cas9‐VQR system

Name of potential off‐target site	Location	Sequence	Guide RNA	Promoters	No. of plants sequenced	No. of modified plants	Mutation rate (%)
VQR‐off‐1	11:18102183‐18102203	AGCGGCGGCGGCGGCGTCAAGA	Unmodified	*2x35S*	11	9	81.82
			Modified	*2x35S*	13	13	100
				*UBI1*	16	16	100
				*ACT1*	28	28	100
VQR‐off‐2	10:15997834:15998455	GGTGGCGGCGGCGGCGTCAGGA	Unmodified	*2x35S*	11	6	54.55
			Modified	*2x35S*	13	12	92.31
				*UBI1*	16	16	100
				*ACT1*	28	28	100
VQR‐off‐3	5:25504329:25504950	GGCGTCGGCGGCGGCGTCATGA	Unmodified	*2x35S*	11	1	9.09
			Modified	*2x35S*	13	0	0
				*UBI1*	16	2	12.50
				*ACT1*	28	1	3.57
VQR‐off‐4	6:13414921:13415542	GGCGGCGGCGGCGCCGTCAAGA	Unmodified	*2x35S*	11	0	0
			Modified	*2x35S*	13	0	0
				*UBI1*	16	0	0
				*ACT1*	28	0	0

The protospacer adjacent motif (PAM) and the mismatch nucleotides are highlighted in blue and red, respectively.

## Discussion

In the present study, by the modification of sgRNAs and replacement of rice strong promoters, we revealed that the CRISPR‐Cas9‐VQR system is efficient in genome editing at sites containing NGA PAMs.

The genome editing using the CRISPR‐Cas9 system requires two components: a Cas9 nuclease to cut the target sequence and a sgRNA that binds to the target sequence. The robustness of the system is highly correlated with the level of expression of both the components (Ma *et al*., 2015b). Previous studies in human cells showed that the mutation of continuous Ts and the extension of the duplex of sgRNA could result in increased sgRNA production, which significantly improves the editing efficiency of the system (Dang *et al*., 2015). We adopted a similar strategy to modify the sgRNA structure and found that it can also improve the efficiency of genome editing in both the CRISPR‐Cas9 and CRISPR‐Cas9‐VQR systems. In addition to modified sgRNA, the rice strong endogenous promoters could further enhance the editing efficiency of the CRISPR‐Cas9‐VQR system, particularly with the *ACT1* promoter that could increase the average editing frequency nearly sixfold compared with the original system. Moreover, the frequency of edited plants containing double or triple mutations increased approximately 15‐fold. Previous studies revealed that the editing efficiency of the VQR variant at NGAA or NGAT PAMs is lower than that at NGAG PAM (Kleinstiver *et al*., 2015). Our study found that the increases in editing efficiency at the sites containing NGAA or NGAT PAMs are more significant than those harbouring NGAG sites, suggesting that the inefficient PAMs might be more sensitive to the expression levels of the VQR variant and sgRNA.

Various studies using the CRISPR‐Cas9 system showed that a high editing efficiency is frequently accompanied by a high frequency of off‐targeting events (Fu *et al*., 2013; Hsu *et al*., 2013; Wang *et al*., 2015b; Zhang *et al*., 2014). We also found that the closer the mismatch to the PAMs, the lower the off‐targeting frequency. Our results demonstrated that a similar pattern exists in the CRISPR‐Cas9‐VQR system. Therefore, the off‐targeting should be taken into account when the efficient CRISPR‐Cas9‐VQR system is used for genome editing.

Collectively, we modified the CRISPR‐Cas9‐VQR system and significantly increased the efficiency of genome editing. The work provided a more powerful system for genome editing at the sites harbouring noncanonical NGA PAMs in plants.

## Experimental procedures

### Modification of sgRNA

The modification used is based on *SK‐gRNA*, which assembled the expression cassette of sgRNAs described in our previous study (Wang *et al*., 2015a). The *SK‐gRNA* digested with two restriction enzymes (*Sal*I and *kpn*I) and the big fragment was purified as the vector. The expression cassette of modified sgRNAs was divided into two small fragments for polymerase chain reaction (PCR) with KOD FX DNA Polymerase (TOYOBO, Japan) and connected into the vector. Modified‐1‐F and Modified‐1‐R were designed for one of two small fragments, whereas Modified‐2‐F and Modified‐2‐R were designed for the remaining fragments (Table S12). The template used was *SK‐gRNA,* and the thermocycler was set for one cycle of 94 °C for 2 min, 32 cycles of 98 °C for 10 s, 58 °C for 30 s, 68 °C for 30 s and one cycle of 68 °C for 5 min, and maintained at 4 °C. The recombination of the vector and two small fragments was conducted with the ClonExpress MultiS One Step Cloning Kit (Vazyme, Nanjing, China).

### Construction of expressing plasmids

The sgRNA‐Cas9 expressing plasmids were constructed using the isocaudamer ligation method (Wang *et al*., 2015a). The sgRNAs of *MOC3* (digested with *Kpn*I/*Sal*I) and *GW2* (digested with *Xho*I/*Bgl*II) were assembled in one pC1300‐Cas9 binary vector (digested with *Kpn*I/*Bam*HI). The sgRNA for VQR variants (digested with *Kpn*I/*Bgl*II) was assembled into pC1300‐*2x35S/UBI1* pro‐VQR binary vector (digested with *Kpn*I/*Bam*HI), respectively. In addition, another sgRNA for VQR variants (digested with *Kpn*I/*Nhe*I) was assembled into pC1300‐*ACT1* pro‐VQR binary vector (digested with *Kpn*I/*Xba*I).

### The generation of transgenic rice

The rice (*Oryza sativa* L. ssp. *japonica*) variety *Nipponbare* was used as the host plant in the present study. The strain EHA105 was used for generation of transgenic rice by the *Agrobacterium*‐mediated method, which was conducted as previously described (Hiei *et al*., 1994).

### Replacement of promoters

The *ACT1* promoter was amplified from pcambia2300Actin vector using ACT1‐F/ACT1‐R primers, whereas the *UBI1* promoter was amplified from the rice genome using UBI1‐F/UBI1‐R primers (Table S12). The promoters were recombined into pC1300‐VQR (digested with *Kpn*I/*Nco*I) by Gibson Assembly.

### PCR amplification of target regions and sequencing

The primers used for PCR amplification are listed in Table S12 with KOD FX DNA Polymerase (TOYOBO, Japan). The product of PCR was sequenced by the Sanger method, and the multiple peaks were decoded by the Degenerate Sequence Decoding (DSD) method (Ma *et al*., 2015a).

## Author Contributions

K.W. and J.L. designed the studies. X.H. and X.M. performed the experiments. Q.L. conducted the bioinformatic analyses. K.W., J.L., X.H. and X.M. wrote the manuscript.

## Competing Financial Interests

The authors declare no conflict of interests.

## Supporting information


**Figure S1** The sequence of *UBI1* promoter.
**Figure S2** The sequence of *ACT1* promoter.
**Figure S3** Off‐target effects of different systems in modified plants. Four potential off‐target sites with one mismatch are detected in all modified plants.
**Table S1** The primers and oligos used to construct the sgRNA.
**Table S2** The results of sequence modification using CRISPR‐ Cas9 system.
**Table S3** Comparison of mutations at *MOC3* and *GW2* sites using unmodified and modified sgRNAs.
**Table S4** Comparison of double mutations at *MOC3* and *GW2* sites using unmodified and modified sgRNAs.
**Table S5** The results of sequence modification by VQR using modified sgRNA and *2x35S* promoter.
**Table S6** Comparison of the mutations in CRISPR‐Cas9‐VQR system using unmodified and modified sgRNAs.
**Table S7** Comparison of double and triple mutations in CRISPR‐Cas9‐VQR system.
**Table S8** The results of sequence modification by VQR using modified sgRNA and *UBI1* promoter.
**Table S9** The results of sequence modification by VQR using modified sgRNA and *ACT1* promoter.
**Table S10** Comparison of the mutations using different promoters in CRISPR‐Cas9‐VQR system.
**Table S11** The results of off‐target with VQR.
**Table S12** The primers used in the study.
