# Supplementary material for: Increasing the efficiency of CRISPR‐Cas9‐VQR precise genome editing in rice
Source: Plant Biotechnol J. 2017 Aug 5;16(1):292–7. doi: 10.1111/pbi.12771 (PMC5785341; doi:10.1111/pbi.12771)
Supplement: Supplementary file 1 — Figure S1 The sequence of UBI1 promoter. Figure S2 The sequence of ACT1 promoter. Figure S3 Off‐target effects of different systems in modified plants. Four potential off‐target sites with one mismatch are detected in all modified plants. Table S1 The primers and oligos used to construct the sgRNA. Table S2 The results of sequence modification using CRISPR‐ Cas9 system. Table S3 Comparison of mutations at MOC3 and GW2 sites using unmodified and modified sgRNAs. Table S4 Comparison of double mutations at MOC3 and GW2 sites using unmodified and modified sgRNAs. Table S5 The results of sequence modification by VQR using modified sgRNA and 2x35S promoter. Table S6 Comparison of the mutations in CRISPR‐Cas9‐VQR system using unmodified and modified sgRNAs. Table S7 Comparison of double and triple mutations in CRISPR‐Cas9‐VQR system. Table S8 The results of sequence modification by VQR using modified sgRNA and UBI1 promoter. Table S9 The results of sequence modification by VQR using modified sgRNA and ACT1 promoter. Table S10 Comparison of the mutations using different promoters in CRISPR‐Cas9‐VQR system. Table S11 The results of off‐target with VQR. Table S12 The primers used in the study. [file PBI-16-292-s001.doc]

**Supporting information**

**Supplementary Fig. 1.** The sequence of *UBI1* promoter

**Supplementary Fig. 2.** The sequence of *ACT1* promoter

**Supplementary Fig. 3.** Off-target effects of different systems in modified plants. Four potential off-target sites with one mismatch are detected in all modified plants.

**Supplementary Table. 1.** The primers and oligos used to construct the sgRNA

**Supplementary Table. 2.** The results of sequence modification using CRISPR- Cas9 system

**Supplementary Table. 3.** Comparison of mutations at *MOC3* and *GW2* sites using unmodified and modified sgRNAs

**Supplementary Table. 4.** Comparison of double mutations at *MOC3* and *GW2* sites using unmodified and modified sgRNAs

**Supplementary Table. 5.** The results of sequence modification by VQR using modified sgRNA and *2x35S* promoter

**Supplementary Table. 6.** Comparison of the mutations in CRISPR-Cas9-VQR system using unmodified and modified sgRNAs

**Supplementary Table. 7.** Comparison of double and triple mutations in CRISPR-Cas9-VQR system

**Supplementary Table. 8.** The results of sequence modification by VQR using modified sgRNA and *UBI1* promoter

**Supplementary Table. 9.** The results of sequence modification by VQR using modified sgRNA and *ACT1* promoter

**Supplementary Table. 10.** Comparison of the mutations using different promoters in CRISPR-Cas9-VQR system

**Supplementary Table. 11.** The results of off-target with VQR.

**Supplementary Table. 12.** The primers used in the study

**Supplementary Fig. 1**

*UBI1* promoter

>gi|937920774:28400831-28402584 *Oryza sativa* Japonica Group DNA, chromosome 6, cultivar: Nipponbare, complete sequence

GTCGACCTGATGATTATTTTGTTGATCATGATTTTCTTTTGGCTATTTGATTTTTTGAAAGATATTTTTTTCCCTGGGAAGACACCTATGGGACGAAGATATTATGTTTCTTATATAGCACCAAACAAATTTAATATATATATATATATATATATATATATATATATATATATATATATATATATATATATATATATATATATATATATATATATATCACATCAGTCTCTGCACAAAGTGCATCCTGGGCTGCTTCAATTATAAAGCCCCATTCACCACATTTGCTAGATAGTCGAAAAGCACCATCAATATTGAGCTTCAGGTATTTTTGGTTGTGTTGTGGTTGGATTGATTCTAATATATACCAAATCAATATAATTCACTACCAAAATATACCATAGCCATCACAACTTTATTAATTTTGGTAGCTTAAGATGGTATATATAATAACCAATTAACAACTGATTCTAATTTTACTACGGCCCAGTATGTACCAATACAAAACAACGAGTATGTTTTCTTCCATCGTAATCGTACACAGTACAAAAAAACCTGGCCAGCCTTTCTTGGGCTGGGGCTCTCTTTCGAAAGGTCACAAAACGTACACGGCAGTAACGCCGCTTCGCTGCGTGTTAACGGCCACCAACCCCGCCGTGAGCAAACGGCATCAGCTTTCCACCTCCTCGATATCTCCGCGGCGCCGTCTGGACCCGCCCCCTTTCCGTTCCTTTCTTTCCTTCTCGCGTTTGCGTGGTGGGGACGGACTCCCCAAACCGCCTCTCCCTCTCTCCTTTCTTTATTTGTCTATATTCTCACTGGGCCCCACCCACCGCACCCCTGGGCCCACTCACGAGTCCCCCCCTCCCCACCTATAAATACCCCACCCCCTCCTCGCCTCTTCCTCCGTCAATCGAACCCCAAAATCGCAGAGAAAAAAAAATCTCCCCTCGAAGCGAAGCGTCGAATCGCCTTCTCAAGGTATGCGATTTTCTGATCCTCTCCGTTCCTCGCGTTTGATTTGATTTCCCGGCCTGTTCGTGATTGTGAGATGTTGTGGTTAGTCTCCGTTTTGCGATCTGTGGTAGATTTGAACAGGTTTAGATGGGGTTCGCGTGGTATGCTGGATCTGTGATTATGAGCGATGCTGTTCGTGGTCCAAGTATTGATTGGTTCGGATCTAGAAGTAGAACTGTGCTAGGGTTGTGATTTGTTCCGATCTGTTCAATTAGTAGGATTTAGTCTCTGTTTTTCTCGTTGATCCAAGTAGCAGCTTCAGGTATATTTTGCTTAGGTTGTTTTTGATTCAGTCCCTCTAGTTGCATAGATTCTACTCTGTTCATGTTTAATCTAAGGGCTGCGTCTTGTTGATTAGTGATTACATAGCATAGCTTTCAGGATATTTTACTTGCTTATGCCTATCTTATCAACTGTTGCACCTGTAAATTCTAGCCTATGTTAATTAACCTGCCTTATGTGCTCTCGGGATAGTGCTAGTAGTTATTGAATCAGTTTGCCGATGGAATTCTAGTAGTTCATAGACCTGCAGATTATTTTTGTGAACTCGAGCACGGTGCGTCTCTCTATTTTGTTAGGTCACTGTTGGTGTTGATAGGTACACTGATGTTATTGTGGTTTAGGTCGTGTATCTAACATATTGGAATAATTTGATTGACTGATTTCTGCTGTACTTGCTTGGTATTGTTATAATTTCATGTTCATAGTTGCTGACCATGCTTCGGTAATTGTGTGTGCAG

Supplementary Fig. 1: The sequence of *UBI1* promoter

**Supplementary Fig. 2**

*ACT1* promoter

>EU155408.1

TAGCTAGCATACTCGAGGTCATTCATATGCTTGAGAAGAGAGTCGGGATAGTCCAAAATAAAACAAAGGTAAGATTACCTGGTCAAAAGTGAAAACATCAGTTAAAAGGTGGTATAAAGTAAAATATCGGTAATAAAAGGTGGCCCAAAGTGAAATTTACTCTTTTCTACTATTATAAAAATTGAGGATGTTTTTGTCGGTACTTTGATACGTCATTTTTGTATGAATTGGTTTTTAAGTTTATTCGCTTTTGGAAATGCATATCTGTATTTGAGTCGGGTTTTAAGTTCGTTTGCTTTTGTAAATACAGAGGGATTTGTATAAGAAATATCTTTAAAAAAACCCATATGCTAATTTGACATAATTTTTGAGAAAAATATATATTCAGGCGAATTCTCACAATGAACAATAATAAGATTAAAATAGCTTTCCCCCGTTGCAGCGCATGGGTATTTTTTCTAGTAAAAATAAAAGATAAACTTAGACTCAAAACATTTACAAAAACAACCCCTAAAGTTCCTAAAGCCCAAAGTGCTATCCACGATCCATAGCAAGCCCAGCCCAACCCAACCCAACCCAACCCACCCCAGTCCAGCCAACTGGACAATAGTCTCCACACCCCCCCACTATCACCGTGAGTTGTCCGCACGCACCGCACGTCTCGCAGCCAAAAAAAAAAAAAGAAAGAAAAAAAAGAAAAAGAAAAAACAGCAGGTGGGTCCGGGTCGTGGGGGCCGGAAACGCGAGGAGGATCGCGAGCCAGCGACGAGGCCGGCCCTCCCTCCGCTTCCAAAGAAACGCCCCCCATCGCCACTATATACATACCCCCCCCTCTCCTCCCATCCCCCCAACCCTACCACCACCACCACCACCACCTCCACCTCCTCCCCCCTCGCTGCCGGACGACGAGCTCCTCCCCCCTCCCCCTCCGCCGCCGCCGCGCCGGTAACCACCCCGCCCCTCTCCTCTTTCTTTCTCCGTTTTTTTTTCCGTCTCGGTCTCGATCTTTGGCCTTGGTAGTTTGGGTGGGCGAGAGGCGGCTTCGTGCGCGCCCAGATCGGTGCGCGGGAGGGGCGGGATCTCGCGGCTGGGGCTCTCGCCGGCGTGGATCCGGCCCGGATCTCGCGGGGAATGGGGCTCTCGGATGTAGATCTGCGATCCGCCGTTGTTGGGGGAGATGATGGGGGGTTTAAAATTTCCGCCATGCTAAACAAGATCAGGAAGAGGGGAAAAGGGCACTATGGTTTATATTTTTATATATTTCTGCTGCTTCGTCAGGCTTAGATGTGCTAGATCTTTCTTTCTTCTTTTTGTGGGTAGAATTTGAATCCCTCAGCATTGTTCATCGGTAGTTTTTCTTTTCATGATTTGTGACAAATGCAGCCTCGTGCGGAGCTTTTTTGTAGGTAGAAGATGGCT

Supplementary Fig. 2: The sequence of *ACT1* promoter

**Supplementary Fig. 3**


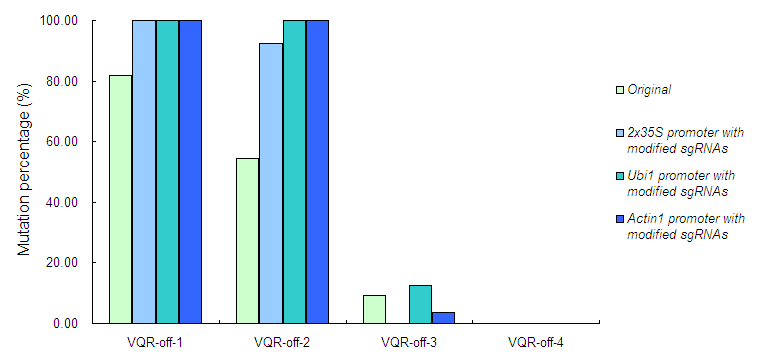


Supplementary Fig. 3: Off-target effects of different systems in modified plants. Four potential off-target sites with one mismatch are detected in all modified plants.

**Supplementary Table. 1** The primers and oligos used to construct the sgRNA

| Target | Location | Target site (5’-3’) | Oligos for customized gRNA construct (5’-3’) | Primers for PCR/RE assay and sequencing (5’-3’) |
| --- | --- | --- | --- | --- |
|  |  | (The PAM is highlighted in red ) |  |  |
| MOC3 | Chr04:33860633-33860655 | GGGAAGAGTGGAAGCGTCTCGGG | g++:GGCAGGGAAGAGTGGAAGCGTCTC g--:AAACGAGACGCTTCCACTCTTCCC | F:TACCTCCTCCAGGTGTGTGT R:ATGGTGTTGGAGGTAGCAGC |
|  |  |  |  |  |
| GW2 | Chr02:8117016-8117037 | ATCAAAGTGTTGCTCAAAAGGG | g++:GGCAATCAAAGTGTTGCTCAAAA g--:AAACTTTTGAGCAACACTTTGAT | F:GTGTGTGGCCGTTATGGAGA R:TGTGTAGGCTGAGCAGTGTG |
|  |  |  |  |  |
| Target-A | Chr05:2247060:2247681 | GGCGGCGGCGGCGGCGTCATGA | g++:GGCAGGCGGCGGCGGCGGCGTCA g--:AAACTGACGCCGCCGCCGCCGCC | F:GTGCCGTGGTTGCATTACC R:AGTCGGTGCCGAGTGGTAG |
|  |  |  |  |  |
| Target-T | Chr01:39790742:39791363 | GGCGGCGGCGGCGGCGTCATGA | g++:GGCAGGCGGCGGCGGCGGCGTCA g--:AAACTGACGCCGCCGCCGCCGCC | F:CTGCCGAAGGTGATCGAAG R:CCAATTGCTAGATCAGTGGTG |
|  |  |  |  |  |
| Target-G | Chr03:9130970:9131591 | GGCGGCGGCGGCGGCGTCATGA | g++:GGCAGGCGGCGGCGGCGGCGTCA g--:AAACTGACGCCGCCGCCGCCGCC | F:CTCCCTTCCCCTCCTCTTC R:CCCATGGCTAGGACAGGAT |
|  |  |  |  |  |

**Supplementary Table. 2** The results of sequence modification using CRISPR- Cas9 system

|  | Unmodified sgRNAs | | Modified sgRNAs | |
| --- | --- | --- | --- | --- |
|  | MOC3 | GW2 | MOC3 | GW2 |
| #1 | Monoallelic mutation | WT | WT | WT |
| #2 | Monoallelic mutation | WT | Chimera with WT | Biallelic mutation |
| #3 | WT | WT | Chimera with WT | WT |
| #4 | WT | WT | Chimera with WT | WT |
| #5 | WT | WT | Chimera without WT | Biallelic mutation |
| #6 | WT | WT | Chimera without WT | Biallelic mutation |
| #7 | WT | WT | WT | WT |
| #8 | Monoallelic mutation | Monoallelic mutation | WT | Monoallelic mutation |
| #9 | Monoallelic mutation | WT | Chimera without WT | Chimera with WT |
| #10 | WT | WT | WT | WT |
| #11 | Monoallelic mutation | WT | WT | WT |
| #12 | Monoallelic mutation | Monoallelic mutation | WT | WT |
| #13 | Biallelic mutation | Monoallelic mutation | WT | WT |
| #14 | Monoallelic mutation | Monoallelic mutation | Biallelic mutation | Chimera without WT |
| #15 | Monoallelic mutation | Monoallelic mutation | Chimera without WT | Monoallelic mutation |
| #16 | Biallelic mutation | Chimera with WT | WT | WT |
| #17 | Monoallelic mutation | Monoallelic mutation | WT | WT |
| #18 | Biallelic mutation | Monoallelic mutation | Chimera without WT | Biallelic mutation |
| #19 | Monoallelic mutation | WT | WT | WT |
| #20 | WT | WT | Biallelic mutation | Monoallelic mutation |
| #21 | WT | WT | Biallelic mutation | Chimera with WT |
| #22 | WT | WT | Biallelic mutation | WT |
| #23 | Monoallelic mutation | Monoallelic mutation | Biallelic mutation | Biallelic mutation |
| #24 | WT | WT | Biallelic mutation | WT |
| #25 | WT | WT | WT | WT |
| #26 | Monoallelic mutation | Monoallelic mutation | WT | WT |
| #27 | WT | WT | WT | WT |
| #28 | Chimera without WT | Monoallelic mutation | WT | WT |
| #29 | Monoallelic mutation | WT | Biallelic mutation | Biallelic mutation |
| #30 | Monoallelic mutation | Monoallelic mutation | Monoallelic mutation | Monoallelic mutation |
| #31 | WT | WT | Biallelic mutation | Chimera with WT |
| #32 | Monoallelic mutation | Chimera without WT | Biallelic mutation | Biallelic mutation |
| #33 | Monoallelic mutation | Monoallelic mutation | WT | WT |
| #34 | Monoallelic mutation | WT | Biallelic mutation | Biallelic mutation |
| #35 | WT | WT | Monoallelic mutation | Monoallelic mutation |
| #36 | Chimera without WT | Monoallelic mutation | WT | WT |
| #37 | Monoallelic mutation | Chimera with WT | Biallelic mutation | Biallelic mutation |
| #38 | WT | WT | Biallelic mutation | Biallelic mutation |
| #39 | WT | WT | WT | WT |
| #40 | WT | WT | Monoallelic mutation | Biallelic mutation |
| #41 | Biallelic mutation | WT | Chimera without WT | Monoallelic mutation |
| #42 | Monoallelic mutation | Monoallelic mutation | WT | WT |
| #43 | Biallelic mutation | Monoallelic mutation | WT | WT |
| #44 | WT | WT | WT | WT |
| #45 | Biallelic mutation | Monoallelic mutation | Chimera without WT | Biallelic mutation |
| #46 | Biallelic mutation | Monoallelic mutation | WT | WT |
| #47 | Monoallelic mutation | Monoallelic mutation | WT | WT |
| #48 | WT | WT | Biallelic mutation | Chimera without WT |
| #49 | WT | Monoallelic mutation | Chimera without WT | Chimera without WT |
| #50 | WT | WT | WT | WT |
| #51 | WT | WT | Monoallelic mutation | Monoallelic mutation |
| #52 | Chimera without WT | WT | Biallelic mutation | Biallelic mutation |
| #53 | Monoallelic mutation | WT | Biallelic mutation | Chimera with WT |
| #54 | Chimera with WT | WT | Chimera without WT | Monoallelic mutation |
| #55 | Monoallelic mutation | Monoallelic mutation | Biallelic mutation | Biallelic mutation |
| #56 | WT | WT | Chimera without WT | Chimera without WT |
| #57 | Monoallelic mutation | WT | WT | Monoallelic mutation |
| #58 | Monoallelic mutation | Monoallelic mutation | Biallelic mutation | Chimera without WT |
| #59 | Monoallelic mutation | Biallelic mutation | WT | WT |
| #60 | WT | WT | Chimera without WT | Biallelic mutation |
| #61 | Monoallelic mutation | WT | Chimera without WT | Chimera without WT |
| #62 | Monoallelic mutation | WT | WT | WT |
| #63 | WT | WT | Biallelic mutation | WT |
| #64 | Chimera without WT | Monoallelic mutation | WT | WT |
| #65 | Monoallelic mutation | WT | WT | WT |
| #66 | Monoallelic mutation | Biallelic mutation | Chimera without WT | Chimera without WT |
| #67 | Chimera with WT | Monoallelic mutation | WT | WT |
| #68 | Monoallelic mutation | WT | Biallelic mutation | Biallelic mutation |
| #69 | WT | WT | Biallelic mutation | Monoallelic mutation |
| #70 | Chimera without WT | Biallelic mutation | Chimera without WT | Chimera without WT |
| #71 | Monoallelic mutation | WT | Monoallelic mutation | WT |
| #72 | WT | WT | Biallelic mutation | Chimera without WT |
| #73 | Biallelic mutation | Monoallelic mutation | WT | WT |
| #74 | Biallelic mutation | WT | Biallelic mutation | WT |
| #75 | Biallelic mutation | WT | Monoallelic mutation | Monoallelic mutation |
| #76 | WT | WT | Monoallelic mutation | WT |
| #77 | Monoallelic mutation | Biallelic mutation | Monoallelic mutation | Monoallelic mutation |
| #78 | Monoallelic mutation | Monoallelic mutation | WT | WT |
| #79 | Monoallelic mutation | Monoallelic mutation | Biallelic mutation | Biallelic mutation |
| #80 | WT | WT | Biallelic mutation | Chimera without WT |
| #81 | Biallelic mutation | Monoallelic mutation | Monoallelic mutation | Monoallelic mutation |
| #82 | Monoallelic mutation | WT | Monoallelic mutation | Biallelic mutation |
| #83 | WT | WT | Chimera with WT | Biallelic mutation |
| #84 | Biallelic mutation | Monoallelic mutation | Monoallelic mutation | Biallelic mutation |
| #85 | Biallelic mutation | Biallelic mutation | Biallelic mutation | Chimera with WT |
| #86 | Biallelic mutation | Monoallelic mutation | Monoallelic mutation | WT |
| #87 | Biallelic mutation | Monoallelic mutation | Chimera without WT | Chimera with WT |
| #88 | Biallelic mutation | Monoallelic mutation | Chimera without WT | Chimera without WT |
| #89 | \ | \ | Chimera without WT | Biallelic mutation |
| #90 | \ | \ | WT | WT |
| #91 | \ | \ | Monoallelic mutation | Monoallelic mutation |
| #92 | \ | \ | WT | WT |

**Supplementary Table. 3** Comparison of mutations at *MOC3* and *GW2* sites using unmodified and modified sgRNAs

| Target gene | Guide RNA | No. of plants detected | No. of modified plants | Mutation rate (%) | No. of mutants | Proportion of mutants (%) |
| --- | --- | --- | --- | --- | --- | --- |
|  |  |  |  |  |  |  |
|  |  |  |  |  |  |  |
| *MOC3* | unmodified | 88 | 58 | 65.91 | 21 | 23.86 |
|  | modified | 92 | 59 | 64.13 | 42 | 45.65 |
| *GW2* | unmodified | 88 | 39 | 44.32 | 6 | 6.82 |
|  | modified | 92 | 52 | 56.52 | 32 | 34.78 |

**Supplementary Table. 4** Comparison of double mutations at *MOC3* and *GW2* sites using unmodified and modified sgRNAs

| Guide RNA | No. of plants detected | No. of *moc3 gw2* | Proportion of *moc3 gw2* (%) |
| --- | --- | --- | --- |
|
|
| unmodified | 88 | 2 | 2.27 |
| modified | 92 | 27 | 29.35 |

**Supplementary Table. 5** The results of sequence modification by VQR using modified sgRNA and *2x35S* promoter

|  | 2x35S promoter and modified sgRNAs | | |
| --- | --- | --- | --- |
|  | Target-A | Target-T | Target-G |
| #1 | WT | WT | WT |
| #2 | WT | WT | WT |
| #3 | WT | WT | WT |
| #4 | WT | WT | WT |
| #5 | WT | WT | Chimera with WT |
| #6 | WT | WT | WT |
| #7 | WT | WT | WT |
| #8 | WT | WT | WT |
| #9 | WT | WT | WT |
| #10 | WT | WT | Monoallelic mutation |
| #11 | WT | WT | Monoallelic mutation |
| #12 | WT | WT | Monoallelic mutation |
| #13 | WT | WT | WT |
| #14 | Monoallelic mutation | WT | Monoallelic mutation |
| #15 | WT | WT | WT |
| #16 | WT | WT | WT |
| #17 | WT | WT | WT |
| #18 | WT | WT | WT |
| #19 | WT | WT | WT |
| #20 | WT | WT | WT |
| #21 | WT | WT | WT |
| #22 | WT | Monoallelic mutation | Monoallelic mutation |
| #23 | Monoallelic mutation | WT | Monoallelic mutation |
| #24 | WT | WT | WT |
| #25 | WT | WT | WT |
| #26 | WT | WT | WT |
| #27 | WT | Monoallelic mutation | Monoallelic mutation |
| #28 | WT | Monoallelic mutation | Monoallelic mutation |
| #29 | WT | WT | WT |
| #30 | WT | WT | WT |
| #31 | WT | Monoallelic mutation | Monoallelic mutation |
| #32 | WT | WT | WT |
| #33 | WT | WT | WT |
| #34 | WT | WT | WT |
| #35 | WT | WT | WT |
| #36 | WT | WT | Monoallelic mutation |
| #37 | WT | WT | WT |
| #38 | WT | WT | WT |
| #39 | WT | WT | WT |
| #40 | WT | WT | WT |
| #41 | WT | WT | WT |
| #42 | WT | WT | Monoallelic mutation |
| #43 | WT | WT | Monoallelic mutation |

**Supplementary Table. 6** Comparison of the mutations in CRISPR-Cas9-VQR system using unmodified and modified sgRNAs

| Target name | Genomic location | Guide RNA | No. of plants detected | No. of modified plants | Mutation rate (%) | No. of biallelic mutations |
| --- | --- | --- | --- | --- | --- | --- |
|  |  |  |  |  |  |  |
| target-A | 5:2247360-2247381 | Unmodified | 49 | 2 | 4.08 | 1 |
|  |  | Modified | 43 | 2 | 4.65 | 0 |
| target-T | 1:39791042-39791063 | Unmodified | 49 | 1 | 2.04 | 0 |
|  |  | Modified | 43 | 4 | 9.30 | 0 |
| target-G | 3:9131270-9131291 | Unmodified | 49 | 9 | 18.37 | 1 |
|  |  | Modified | 43 | 13 | 30.23 | 0 |

**Supplementary Table. 7** Comparison of double and triple mutations in CRISPR-Cas9-VQR system

| CRIPSR-VQR system | No. of plants detected | No. of double mutation | Proportion of double mutations (%) | No. of triple mutation | Proportion of triple mutations (%) |
| --- | --- | --- | --- | --- | --- |
|
|
| Original | 49 | 1 | 2.04 | 0 | 0 |
| *2x35S* pro and modified sgRNA | *43* | 6 | 13.95 | 0 | 0 |
| *UBI1* pro and modified sgRNA | *36* | 5 | 13.89 | 2 | 5.56 |
| *ACT1* pro and modified sgRNA | *36* | 7 | 19.44 | 4 | 11.11 |

**Supplementary Table. 8** The results of sequence modification by VQR using modified sgRNA and *UBI1* promoter

|  | *UBI1* promoter and modified sgRNAs | | |
| --- | --- | --- | --- |
|  | Target-A | Target-T | Target-G |
| #1 | WT | WT | WT |
| #2 | WT | WT | WT |
| #3 | WT | WT | Monoallelic mutation |
| #4 | WT | WT | WT |
| #5 | WT | WT | WT |
| #6 | Monoallelic mutation | WT | Biallelic mutation |
| #7 | WT | WT | WT |
| #8 | WT | WT | WT |
| #9 | WT | WT | WT |
| #10 | WT | Monoallelic mutation | Biallelic mutation |
| #11 | WT | Biallelic mutation | Biallelic mutation |
| #12 | Monoallelic mutation | WT | WT |
| #13 | WT | WT | Chimera with WT |
| #14 | Monoallelic mutation | WT | Biallelic mutation |
| #15 | Monoallelic mutation | WT | WT |
| #16 | WT | WT | WT |
| #17 | Monoallelic mutation | Monoallelic mutation | Biallelic mutation |
| #18 | WT | WT | WT |
| #19 | WT | WT | WT |
| #20 | WT | WT | WT |
| #21 | WT | WT | WT |
| #22 | WT | WT | WT |
| #23 | Monoallelic mutation | WT | Monoallelic mutation |
| #24 | Monoallelic mutation | Monoallelic mutation | Biallelic mutation |
| #25 | WT | WT | WT |
| #26 | WT | WT | WT |
| #27 | WT | WT | WT |
| #28 | WT | WT | WT |
| #29 | WT | WT | Monoallelic mutation |
| #30 | WT | WT | WT |
| #31 | WT | WT | Biallelic mutation |
| #32 | WT | WT | Chimera with WT |
| #33 | WT | WT | Monoallelic mutation |
| #34 | WT | WT | WT |
| #35 | WT | WT | Biallelic mutation |
| #36 | WT | WT | WT |

**Supplementary Table. 9** The results of sequence modification by VQR using modified sgRNA and *ACT1* promoter

|  | *ACT1* promoter and modified sgRNAs | | |
| --- | --- | --- | --- |
|  | Target-A | Target-T | Target-G |
| #1 | WT | WT | Biallelic mutation |
| #2 | Monoallelic mutation | WT | Biallelic mutation |
| #3 | WT | WT | Chimera with WT |
| #4 | Monoallelic mutation | WT | Chimera with WT |
| #5 | WT | WT | Biallelic mutation |
| #6 | WT | WT | Biallelic mutation |
| #7 | WT | Monoallelic mutation | Chimera with WT |
| #8 | WT | WT | Monoallelic mutation |
| #9 | WT | WT | Biallelic mutation |
| #10 | Monoallelic mutation | WT | Biallelic mutation |
| #11 | WT | WT | Biallelic mutation |
| #12 | WT | WT | WT |
| #13 | Monoallelic mutation | WT | Monoallelic mutation |
| #14 | WT | WT | WT |
| #15 | WT | WT | Biallelic mutation |
| #16 | Monoallelic mutation | Monoallelic mutation | Biallelic mutation |
| #17 | WT | WT | WT |
| #18 | WT | WT | WT |
| #19 | WT | WT | Biallelic mutation |
| #20 | WT | WT | WT |
| #21 | WT | WT | Biallelic mutation |
| #22 | WT | WT | Monoallelic mutation |
| #23 | WT | WT | Monoallelic mutation |
| #24 | WT | WT | Biallelic mutation |
| #25 | WT | WT | Monoallelic mutation |
| #26 | WT | WT | Biallelic mutation |
| #27 | WT | WT | WT |
| #28 | WT | WT | Chimera with WT |
| #29 | WT | WT | Biallelic mutation |
| #30 | WT | Monoallelic mutation | Biallelic mutation |
| #31 | Biallelic mutation | Monoallelic mutation | Biallelic mutation |
| #32 | Chimera with WT | Monoallelic mutation | Biallelic mutation |
| #33 | Monoallelic mutation | Monoallelic mutation | Biallelic mutation |
| #34 | WT | WT | WT |
| #35 | WT | WT | WT |
| #36 | Monoallelic mutation | WT | Biallelic mutation |

**Supplementary Table. 10** Comparison of the mutations using different promoters in CRISPR-Cas9-VQR system

| Target name | Genomic location | System | No. of plants detected | No. of modified plants | Mutation rate (%) | No. of biallelic mutations | Proportion of biallelic mutations (%) |
| --- | --- | --- | --- | --- | --- | --- | --- |
|  |  |  |  |  |  |  |  |
| target-A | 5:2247360-2247381 | Original | 49 | 2 | 4.08 | 1 | 2.04 |
|  |  | *UBI1* pro and modified sgRNA | 36 | 7 | 19.44 | 0 | 0 |
|  |  | *ACT1* pro and modified sgRNA | 36 | 9 | 25.00 | 1 | 2.78 |
| target-T | 1:39791042-39791063 | Original | 49 | 1 | 2.04 | 0 | 0 |
|  |  | *UBI1* pro and modified sgRNA | 36 | 4 | 11.11 | 1 | 2.78 |
|  |  | *ACT1* pro and modified sgRNA | 36 | 6 | 16.67 | 0 | 0 |
| target-G | 3:9131270-9131291 | Original | 49 | 9 | 18.37 | 1 | 2.04 |
|  |  | *UBI1* pro and modified sgRNA | 36 | 14 | 38.89 | 8 | 22.22 |
|  |  | *ACT1* pro and modified sgRNA | 36 | 28 | 77.78 | 19 | 52.78 |

**Supplementary Table. 11** The results of off-target with VQR

|  | Original | | | | Modified sgRNA and 2x35S promoter | | | | Modified sgRNA and UBI1 promoter | | | | Modified sgRNA and ACT1 promoter | | | |
| --- | --- | --- | --- | --- | --- | --- | --- | --- | --- | --- | --- | --- | --- | --- | --- | --- |
|  | Off-1 | Off-2 | Off-3 | Off-4 | Off-1 | Off-2 | Off-3 | Off-4 | Off-1 | Off-2 | Off-3 | Off-4 | Off-1 | Off-2 | Off-3 | Off-4 |
| #1 | M | WT | WT | WT | M | M | WT | WT | M | M | WT | WT | M | M | WT | WT |
| #2 | M | M | M | WT | M | WT | WT | WT | M | M | WT | WT | M | M | WT | WT |
| #3 | M | M | WT | WT | M | M | WT | WT | M | M | WT | WT | M | M | WT | WT |
| #4 | M | M | WT | WT | M | M | WT | WT | M | M | WT | WT | M | M | WT | WT |
| #5 | M | WT | WT | WT | M | M | WT | WT | M | M | WT | WT | M | M | M | WT |
| #6 | M | WT | WT | WT | M | M | WT | WT | M | M | WT | WT | M | M | WT | WT |
| #7 | WT | WT | WT | WT | M | M | WT | WT | M | M | WT | WT | M | M | WT | WT |
| #8 | WT | WT | WT | WT | M | M | WT | WT | M | M | WT | WT | M | M | WT | WT |
| #9 | M | M | WT | WT | M | M | WT | WT | M | M | WT | WT | M | M | WT | WT |
| #10 | M | M | WT | WT | M | M | WT | WT | M | M | M | WT | M | M | WT | WT |
| #11 | M | M | WT | WT | M | M | WT | WT | M | M | WT | WT | M | M | WT | WT |
| #12 | \ | \ | \ | \ | M | M | WT | WT | M | M | M | WT | M | M | WT | WT |
| #13 | \ | \ | \ | \ | M | M | WT | WT | M | M | WT | WT | M | M | WT | WT |
| #14 | \ | \ | \ | \ | \ | \ | \ | \ | M | M | WT | WT | M | M | WT | WT |
| #15 | \ | \ | \ | \ | \ | \ | \ | \ | M | M | WT | WT | M | M | WT | WT |
| #16 | \ | \ | \ | \ | \ | \ | \ | \ | M | M | WT | WT | M | M | WT | WT |
| #17 | \ | \ | \ | \ | \ | \ | \ | \ | \ | \ | \ | \ | M | M | WT | WT |
| #18 | \ | \ | \ | \ | \ | \ | \ | \ | \ | \ | \ | \ | M | M | WT | WT |
| #19 | \ | \ | \ | \ | \ | \ | \ | \ | \ | \ | \ | \ | M | M | WT | WT |
| #20 | \ | \ | \ | \ | \ | \ | \ | \ | \ | \ | \ | \ | M | M | WT | WT |
| #21 | \ | \ | \ | \ | \ | \ | \ | \ | \ | \ | \ | \ | M | M | WT | WT |
| #22 | \ | \ | \ | \ | \ | \ | \ | \ | \ | \ | \ | \ | M | M | WT | WT |
| #23 | \ | \ | \ | \ | \ | \ | \ | \ | \ | \ | \ | \ | M | M | WT | WT |
| #24 | \ | \ | \ | \ | \ | \ | \ | \ | \ | \ | \ | \ | M | M | WT | WT |
| #25 | \ | \ | \ | \ | \ | \ | \ | \ | \ | \ | \ | \ | M | M | WT | WT |
| #26 | \ | \ | \ | \ | \ | \ | \ | \ | \ | \ | \ | \ | M | M | WT | WT |
| #27 | \ | \ | \ | \ | \ | \ | \ | \ | \ | \ | \ | \ | M | M | WT | WT |
| #28 | \ | \ | \ | \ | \ | \ | \ | \ | \ | \ | \ | \ | M | M | WT | WT |

**Note:** “M” indicate the site was modified in this plant

**Supplementary Table. 12** The primers used in the study

| **Primer name** | **Sequence (5'-3')** |
| --- | --- |
|
| MOC3-F | TACCTCCTCCAGGTGTGTGT |
| MOC3-R | ATGGTGTTGGAGGTAGCAGC |
| GW2-F | GTGTGTGGCCGTTATGGAGA |
| GW2-R | TGTGTAGGCTGAGCAGTGTG |
| Target-A-F | GTGCCGTGGTTGCATTACC |
| Target-A-R | AGTCGGTGCCGAGTGGTAG |
| Target-T-F | CTGCCGAAGGTGATCGAAG |
| Target-T-R | CCAATTGCTAGATCAGTGGTG |
| Target-G-F | CTCCCTTCCCCTCCTCTTC |
| Target-G-R | CCCATGGCTAGGACAGGAT |
| Modified-1-F | GCGGCCGCAGATCTGCTAGC |
| Modified-1-R | GCTATGCTGTTTCCAGCATAGCTCTGAAACGTGTGCAGGTGTTGTG |
| Modified-2-F | TATGCTGGAAACAGCATAGCAAGTTGAAATAAGGCTAGTCCGTTAT |
| Modified-2-R | ACTCACTATAGGGCGAATTG |
| ACT1-F | ATGATTACGAATTCGAGCTCGGTACCAATCTAGAGTCATTCATATGCTTGAGAA |
| ACT1-R | CTCTTCTTCTTAGGGGCCATTCTTCTACCTACAAAAAAGC |
| UBI1-F | GAATTCGAGCTCGGTACCAAGGATCCGTCGACCTGATGATTATTTT |
| UBI1-R | CTCTTCTTCTTAGGGGCCATCTGCACACACAATTACCGAA |
| VQR-OFF-1-F | GCGACCTTCTCCAACCTCTA |
| VQR-OFF-1-R | ATCTGTTGATGTCCCCTCCG |
| VQR-OFF-2-F | CAGGGGAGCTCTGCATCAG |
| VQR-OFF-2-R | ACATCCACCACAGCGTTTTAC |
| VQR-OFF-3-F | TCATCCCAAGAGCGTCCG |
| VQR-OFF-3-R | TGCACAGTGTTCGAATCGTC |
| VQR-OFF-4-F | CCTCCAGGCTCGTCAACC |
| VQR-OFF-4-R | AAGAAGAGGAAGAGCGCCAC |
